# Converting ginsenosides from stems and leaves of *Panax notoginseng* by microwave processing and improving their anticoagulant and anticancer activities

**DOI:** 10.1039/c8ra08021f

**Published:** 2018-12-04

**Authors:** Yuan Qu, Hui-Ying Liu, Xiao-Xi Guo, Yan Luo, Cheng-Xiao Wang, Jiang-Hua He, Tian-Rui Xu, Ye Yang, Xiu-Ming Cui

**Affiliations:** Yunnan Provincial Key Laboratory of Panax notoginseng, Key Laboratory of Panax notoginseng Resources Sustainable Development and Utilization of State Administration of Traditional Chinese Medicine, University Based Provincial Key Laboratory of Screening and Utilization of Targeted Drugs, Faculty of Life Science and Technology, Kunming University of Science and Technology No. 727 South Jingming Rd., Chenggong District Kunming 650500 China yangyekm@163.com sanqi37@vip.sina.com +86-183-8715-6001 +86-183-8718-6037; College of Materials and Chemical Engineering, Chongqing University of Arts and Science Chongqing 402160 China

## Abstract

A microwave processing technology was applied to degrade saponins from the stems and leaves of *Panax notoginseng*. Six transformation products (1–6), named 20(*S*)-ginsenoside Rg_3_ (1), 20(*R*)-ginsenoside Rg_3_ (2), notoginsenoside SFt_3_ (3), ginsenoside Rk_1_ (4), ginsenoside Rg_5_ (5), and 20(*S*)-ginsenoside Rh_2_ (6) were isolated and identified from a microwave processed extract of the stems and leaves of *P. notoginseng* (MEL). This transformation method was also applied for producing the minor ginsenosides in flowers, seeds and pedicels of *P. notoginseng*. The extract and compounds 1–6 in MEL were evaluated *in vitro* for anticancer and anticoagulant activities. The results showed that the MEL extract and transformation products had outstanding inhibitory activities against human cervical cancer Hela and lung cancer A549 cells. The strongest inhibitory effect was observed for 20(*S*)-Rh_2_ (6) with an IC_50_ value of 8.23 μM in Hela cells. Moreover, the results showed that the MEL significantly prolonged prothrombin time in a concentration-dependent manner. The anticoagulant effect of the MEL improved with the increased contents of Rk_1_, Rg_5_, and SFt_3_.

## Abstract 2

A microwave processing technology was used to produce anticoagulant and anticancer compounds from the stems and leaves of *Panax notoginseng*.

## Graphical abstract

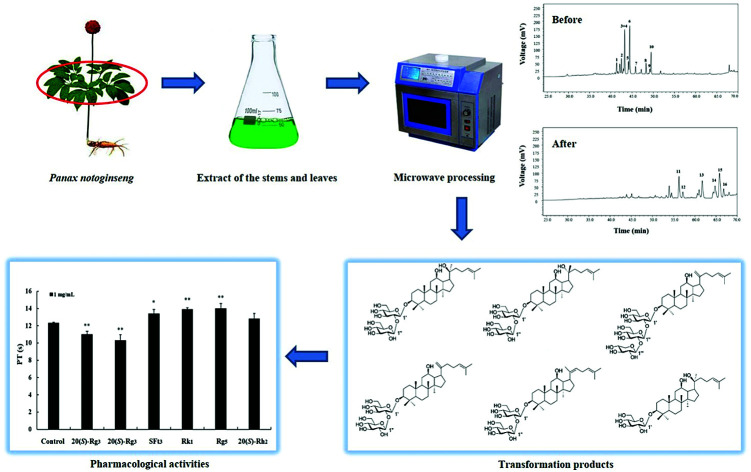

## 1 Introduction

*Panax notoginseng* (Burk.) F. H. Chen has been widely used as a herbal medicine in China. It was recorded in the Compendium of Materia Medica that the stems and leaves of *P. notoginseng* (PNL) were traditionally used to treat bone fractures, eliminate swelling, and stop bleeding. The PNL is also used as a functional food in folk medicine, as PNL is rich in nutrients and characterized by high protein, high dietary fiber, and low fat.^1^ Fresh leaves are suitable for eating as a vegetable and dried as a tea.^2^

**Fig. 1:**
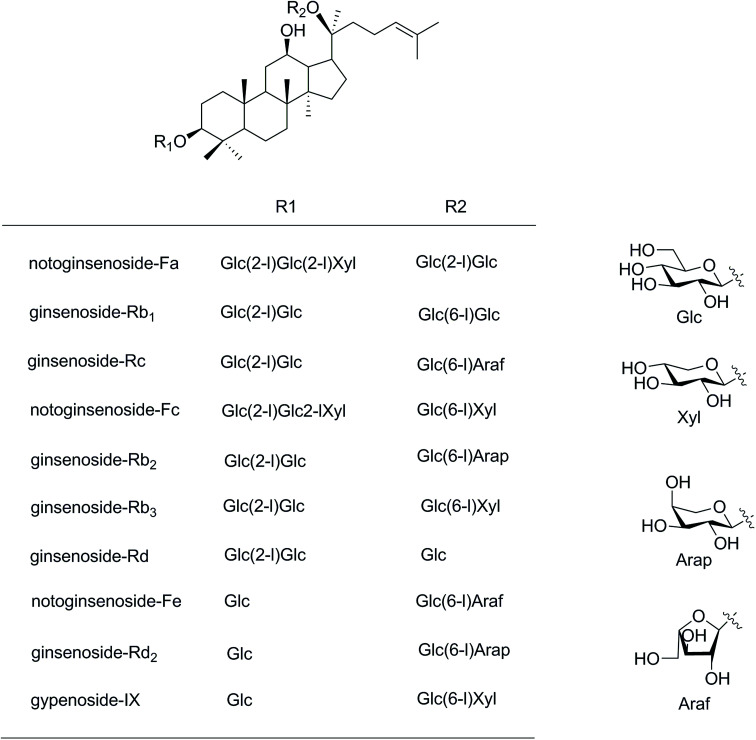
Structures of saponins in the stems and leaves of *Panax notoginseng*. Glc, d-glucopyranosyl; Xyl, d-xylopyranosyl; Ara(f), l-arabinofuranosyl; Ara(p), l-arabinopyranosyl.

More than 40 chemical constituents have been isolated from the PNL. The main components are protopanaxadiol type saponins, such as notoginsenosides Fa, Fc, and Fe, and the ginsenosides Rb_1_, Rc, Rb_2_, and Rb_3_ (Fig. 1).^3^ The saponins have gained prominence owing to their potential pharmaceutical values of analgesic, hypolipidemic, anticancer, anti-arrhythmic, anticoagulatory, anti-inflammatory, and anti-aging activities.^4,5^ The interesting structures are less-polar saponins losing some glycosyl moieties such as 20(*S*/*R*)-ginsenosides Rg_3_ and Rh_2_ because of more effective anticancer activities.^6,7^ The Shenyi capsule containing ginsenoside Rg_3_ was clinically applied for treating non-small cell lung cancer in China.^8^ The common methods used for producing less-polar saponins are acid–base degradation,^9^ enzymatic degradation,^10^ microbial transformation,^11^ and steam processing.^12^ At present, microwave processing method as a novel “green” solvent extraction technology is developed to increase the content of minor ginsenosides from white ginseng.^13,14^ This green extraction process is often used for extracting natural products owing to a faster extraction rate, less consumption of organic solvent, and lower costs of sample preparation,^15,16^ but more rarely in the case of transformation. The heat processing by microwave method for producing the minor ginsenosides gained high yields within about 60 minutes.^13^ The microwave time was much shorter than used in the traditional heating method (over 20 hours).^12^

**Fig. 2:**
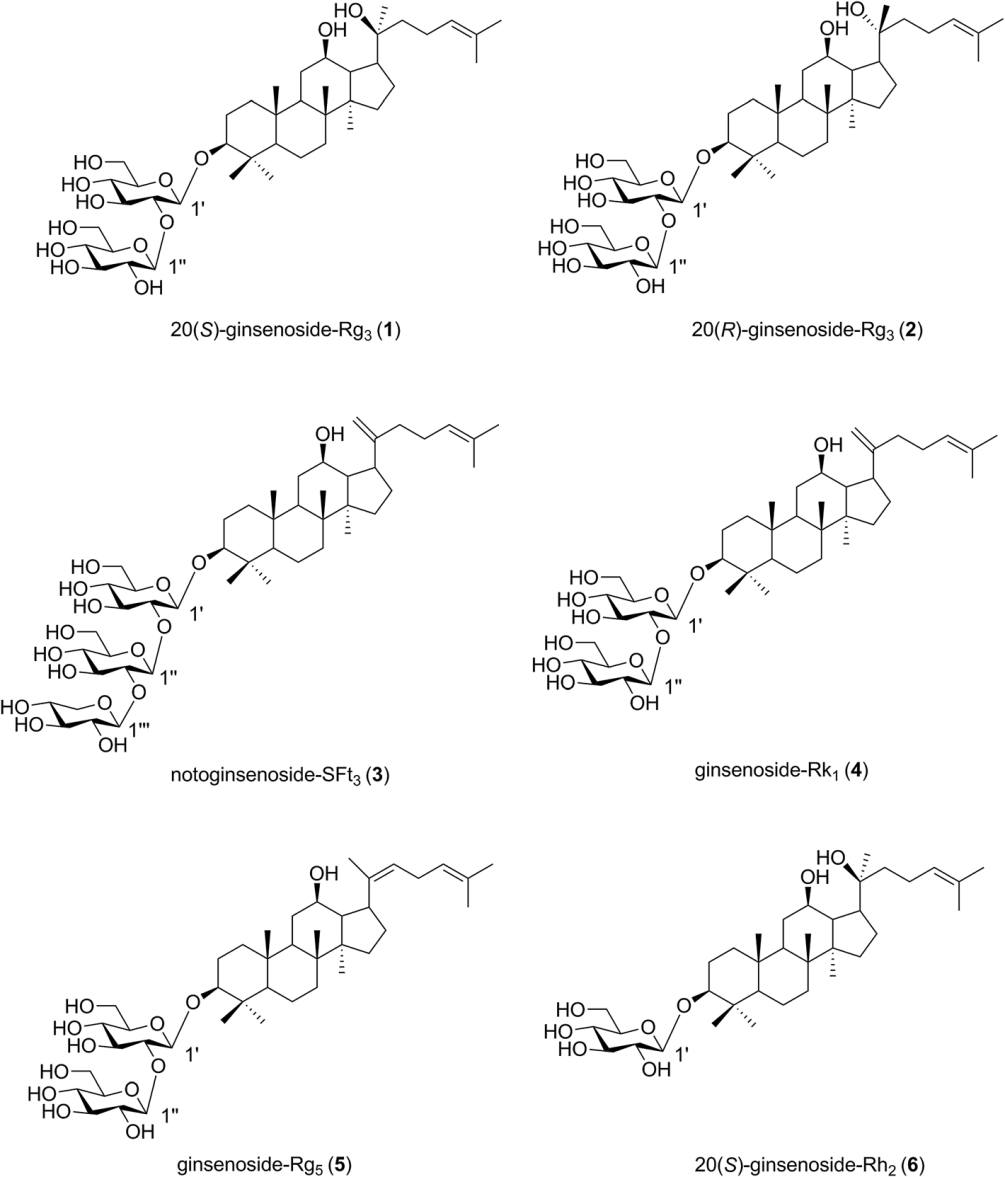
Structures of transformation products 1–6 isolated from microwave processing extract of the stems and leaves of *Panax notoginseng*.

We applied the microwave processing method to accelerate the transformation of minor ginsenosides from the roots of *P. notoginseng* and determined the optimal conversion conditions using response surface methodology.^17^ The roots of *P. notoginseng* are traditionally used as medicine, but a huge amount of PNL is often considered waste. In this study, the microwave method was applied to effectively obtain a microwave processing extract from stems and leaves of *P. notoginseng* (MEL). Six known compounds (1–6) were isolated from the MEL and identified by nuclear magnetic resonance spectroscopic data (Fig. 2). Moreover, the anticancer and anticoagulant effects of the MEL and the transformation products (1–6) were evaluated during microwave processing *in vitro*.

## 2 Materials and methods

### 2.1 Materials and reagents

The different parts of *P. notoginseng* (including stems and leaves, flowers, fruits, and pedicels) were purchased from the wenshan market in Yunnan province of China in October 2016. The samples were identified by Dr Xiuming Cui of Kunming University of Science and Technology. The nuclear magnetic resonance spectra (NMR) was recorded by a Bruker Avance III 500 spectrometer (Bruker Corporation, Berlin, Germany). Formic acid (HPLC grade), acetonitrile (HPLC grade), dimethyl sulfoxide (DMSO), 3-[4,5-dimethylthiazol-2-yl]-2,5-diphenyl-tetrazolium bromide (MTT), penicillin, streptomycin, l-glutamine and methanol were purchased from Sigma-Aldrich (St. Louis., MO, USA). Fetal bovine serum (FBS) and trypsin were purchased from Gibco BRL (Gland Island, NY, USA). All the other reagents and solvents were of analytical grade.

### 2.2 Preparation of processed PNL using the microwave processing method

The dried stems and leaves of *P. notoginseng* (PNL, 50 g) were powdered and ultrasonically extracted with 70% aqueous MeOH, three times for 45 min. After filtration and concentration, the extract of stems and leaves of *P. notoginseng* (EL, 11.25 g) was obtained with a yield of 22.5%. In the same way, the yields of the extracts of flowers (EF), seeds (ES), and pedicels (EP) were 36.36%, 6.48% and 25.48%, respectively.

The extract samples from different parts of *P. notoginseng* (EL, EF, ES and EP) was treated using microwave processing method as reported previously.^17^ 160 mg of the extract was added to the water (10 mL) in a microwave extraction system (model no: MDS-6G) manufactured by Sineo microwave chemical technology Co. Ltd. (Shanghai, China). Considering the influence of microwave temperature, microwave power and time, the temperature ranged from 60 °C to 180 °C, microwave power ranged from 300 W to 1000 W, and time ranged from 5 min to 50 min. After microwave treatment, the sample solution was centrifuged and the supernatant was added to the water for 10 mL. The obtained samples were filtered through a 0.45 μm filter membrane for HPLC analysis. The yields of transformation products were obtained from HPLC data.Yield (%) = *M*_g_ (g)/*M*_s_ (g)*M*_g_ is the content of transformation products and *M*_s_ is total amount of the sample.

### 2.3 Identification of MEL from the stems and leaves of *P. notoginseng* during microwave processing method by UHPLC-ESI-Q-TOF-MS

The EL extract (10 g) was processed at a temperature of 150 °C and microwave power of 500 W for 20 min. After removal of the solvent, microwave processing extract of stems and leaves of *P. notoginseng* (MEL, 9.04 g) was obtained with a yield of 90.4%. The composition of MEL was performed on an Agilent 1290 Infinity II LC system (UHPLC) coupled to an Agilent 6530 quadrupole time-of-flight mass spectrometry system (Q-TOF/MS) (Agilent Technologies, California, America). Samples were separated on an Agilent Poroshell 120 EC-C18 column (4.6 mm × 75 mm, 2.7 ìm) at a rate of 0.6 mL min^−1^ using an Agilent 1290 Infinity II LC system. The mobile phases consisted of 0.1% (v/v) formic acid water (A) and 0.1% (v/v) formic acid acetonitrile (B) in the gradient program: 0–10 min, 10–25% (B); 10–50 min, 25–40% (B); 50–65 min, 40–60% (B); 65–70 min, 60–100% (B). For MS detector performed on a Q-TOF/MS, mass spectrometry conditions of electrospray ionization source (ESI) in positive ion detection mode were as follows: capillary voltage 4000 V; gas temperature, 350 °C; during gas N_2_ flow rate, 10 L min^−1^; pressure of nebulizer, 35 psi. The mass analyzer scanned over *m*/*z* 100–3000 Da.

### 2.4 Purification of transformation products (1–6)

5 g of the MEL extract was subjected to silica gel column chromatography eluted stepwise with MeOH/CHCl_3_ (2 : 98, 5 : 95, 7 : 93, 10 : 90, 15 : 85, 20 : 80, 25 : 75, 30 : 70, 40 : 60, v/v) to afford 5 fractions (Fr. 1–5). Fr. 1 and Fr. 2 were purified by recrystallization to yield compound 1 (126.2 mg) and compound 2 (60 mg), respectively. Fr. 3 was subjected to pre-HPLC [YMC-Pack ODS-A (*ϕ*10 × 250 mm), 2 mL min^−1^ flow rate, MeCN (B)/H_2_O (A) (0–20 min, 40–50% B; 20–65 min, 50–50% B) mobile phase] to give compound 3 (12.8 mg, *t*_R_ = 54.0 min). Fr. 4 was successively purified by pre-HPLC [YMC-Pack ODS-A (ϕ10 × 250 mm), 2 mL min^−1^ flow rate, MeCN (B)/H_2_O (A) (0–40 min, 50–55% B; 40–60 min, 55–55% B) mobile phase] to yield compounds 4 (9.8 mg, *t*_R_ = 54.2 min) and 5 (40.5 mg, *t*_R_ = 58.4 min). Compounds 6 (11.6 mg, *t*_R_ = 58.5 min) was obtained from Fr. 5 separated by pre-HPLC [YMC-Pack ODS-A (*ϕ*10 × 250 mm), 2 mL min^−1^ flow rate, MeCN/H_2_O (55 : 45) mobile phase].

#### 2.4.1 20(*S*)-ginsenoside Rg_3_ (1)

White amorphous powder; HRESIMS *m*/*z* 807.4663 [M + Na]^+^. ^1^H-NMR (C_5_D_5_N, 500 MHz) *δ*: 0.94 (3H, s, H-18), 0.82 (3H, s, H-19), 1.40 (1H, s, H-21), 1.60 (3H, s, H-26), 1.60 (3H, s, H-27), 1.30 (3H, s, H-28), 1.11 (3H, s, H-29), 0.99 (3H, s, H-30), 4.96 (1H, d, *J* = 7.8 Hz, Glc-I-H-1′), 5.38 (1H, d, *J* = 7.7 Hz, Glc-II-H-1′′). ^13^C-NMR (C_5_D_5_N, 125 MHz) *δ*: 39.5(C-1), 27.1(C-2), 89.3(C-3), 40.1(C-4), 56.7(C-5), 18.8(C-6), 35.5(C-7), 40.4(C-8), 50.8(C-9), 37.3(C-10), 32.4(C-11), 71.4(C-12), 48.9(C-13), 52.1(C-14), 31.7(C-15), 27.2(C-16), 55.2(C-17), 16.2(C-18), 16.7(C-19), 73.3(C-20), 27.5(C-21), 36.3(C-22), 23.4(C-23), 126.7(C-24), 131.1(C-25), 26.2(C-26), 17.4(C-27), 28.5(C-28), 17.0(C-29), 18.1(C-30), 105.5 (Glc-I-C-1′), 83.9 (C-2′), 78.4 (C-3′), 72.1 (C-4′), 78.7 (C-5′), 63.2 (C-6′), 106.5 (Glc-II-C-1′′), 77.5 (C-2′′), 78.7 (C-3′′), 72.0 (C-4′′), 78.5 (C-5′′), 63.1 (C-6′′). The structure of compound 1 was identified as 20(*S*)-ginsenoside Rg_3_ by comparing the NMR data and MS data with reported data.^18^

#### 2.4.2 20(*R*)-ginsenoside Rg_3_ (2)

White amorphous powder; HRESIMS *m*/*z* 807.4652 [M + Na]^+^. ^1^H-NMR (C_5_D_5_N, 500 MHz) *δ*: 1.01 (3H, s, H-18), 0.82 (3H, s, H-19), 1.39 (1H, s, H-21), 1.69 (3H, s, H-26), 1.65 (3H, s, H-27), 1.30 (3H, s, H-28), 1.11 (3H, s, H-29), 0.99 (3H, s, H-30), 4.94 (1H, d, *J* = 7.8 Hz, Glc-I-H-1′), 5.38 (1H, d, *J* = 7.7 Hz, Glc-II-H-1′′). ^13^C-NMR (C_5_D_5_N, 125 MHz) *δ*: 39.1(C-1), 26.6(C-2), 88.9(C-3), 39.7(C-4), 56.4(C-5), 18.4(C-6), 35.1(C-7), 39.9(C-8), 50.3(C-9), 36.9(C-10), 31.3(C-11), 70.9(C-12), 49.2(C-13), 51.8(C-14), 32.2(C-15), 26.7(C-16), 50.6(C-17), 15.8(C-18), 16.4(C-19), 73.0(C-20), 22.8(C-21), 43.2(C-22), 22.6(C-23), 126.0(C-24), 130.8(C-25), 25.9(C-26), 17.3(C-27), 28.2(C-28), 16.6(C-29), 17.7(C-30), 105.2 (Glc-I-C-1′), 83.5 (C-2′), 78.0 (C-3′), 71.6 (C-4′), 78.3 (C-5′), 62.9 (C-6′), 106.1 (Glc-II-C-1′′), 77.2 (C-2′′), 78.4 (C-3′′), 71.6 (C-4′′), 78.2 (C-5′′), 62.7 (C-6′′). The structure of compound 2 was identified as 20(*R*)-ginsenoside Rg_3_ by comparing the NMR data and MS data with reported data.^18^

#### 2.4.3 Notoginsenoside SFt_3_ (3)

White amorphous powder; HRESIMS *m*/*z* 921.4989 [M + Na]^+^. ^1^H-NMR (C_5_D_5_N, 500 MHz) *δ*: 0.95 (3H, s, H-18), 0.79 (3H, s, H-19), 5.14 (1H, s, H-21), 4.90 (1H, s, H-21), 1.65 (3H, s, H-26), 1.59 (3H, s, H-27), 1.28 (3H, s, H-28), 1.10 (3H, s, H-29), 1.00 (3H, s, H-30), 4.94 (1H, d, *J* = 7.8 Hz, Glc-I-H-1′), 5.52 (1H, d, *J* = 7.7 Hz, Glc-II-H-1′′), 5.41 (1H, d, *J* = 6.7 Hz, Xyl-H-1′′′). ^13^C-NMR (C_5_D_5_N, 125 MHz) *δ*: 39.8(C-1), 26.8(C-2), 88.8(C-3), 39.3(C-4), 56.4(C-5), 18.5(C-6), 35.4(C-7), 40.3(C-8), 48.3(C-9), 37.1(C-10), 32.7(C-11), 72.4(C-12), 52.5(C-13), 51.2(C-14), 32.8(C-15), 30.8(C-16), 50.8(C-17), 16.6(C-18), 15.9(C-19), 155.6(C-20), 108.2(C-21), 33.9(C-22), 27.1(C-23), 125.3(C-24), 131.2(C-25), 25.8(C-26), 17.8(C-27), 28.1(C-28), 16.7(C-29), 17.0(C-30), 104.8 (Glc-I-C-1′), 83.0 (C-2′), 78.7 (C-3′), 71.1 (C-4′), 78.3 (C-5′), 63.0 (C-6′), 103.2 (Glc-II-C-1′′), 84.5 (C-2′′), 78.0 (C-3′′), 71.8 (C-4′′), 77.9 (C-5′′), 62.8 (C-6′′), 106.5 (Xyl-C-1′′′), 76.0 (C-2′′′), 77.8 (C-3′′′), 70.8 (C-4′′′), 67.5 (C-5′′′). The structure of compound 3 was identified as notoginsenoside SFt_3_ by comparing the NMR data and MS data with reported data.^19^

#### 2.4.4 Ginsenoside Rk_1_ (4)

White amorphous powder; HRESIMS *m*/*z* 789.4595 [M + Na]^+^. ^1^H-NMR (C_5_D_5_N, 500 MHz) *δ*: 1.00 (3H, s, H-18), 0.80 (3H, s, H-19), 5.14 (1H, s, H-21), 4.90 (1H, s, H-21), 1.65 (3H, s, H-26), 1.59 (3H, s, H-27), 1.29 (3H, s, H-28), 1.10 (3H, s, H-29), 0.95 (3H, s, H-30), 4.93 (1H, d, *J* = 7.8 Hz, Glc-I-H-1′), 5.37 (1H, d, *J* = 7.7 Hz, Glc-II-H-1′′). ^13^C-NMR (C_5_D_5_N, 125 MHz) *δ*: 39.6(C-1), 27.2(C-2), 89.2(C-3), 40.1(C-4), 56.7(C-5), 18.8(C-6), 35.7(C-7), 40.5(C-8), 48.6(C-9), 37.3(C-10), 33.1(C-11), 72.8(C-12), 52.9(C-13), 51.5(C-14), 32.9(C-15), 31.2(C-16), 51.2(C-17), 16.8(C-18), 16.1(C-19), 155.9(C-20), 108.5(C-21), 34.2(C-22), 27.4(C-23), 125.8(C-24), 131.5(C-25), 26.1(C-26), 18.1(C-27), 28.4(C-28), 17.0(C-29), 17.3(C-30), 105.5 (Glc-I-C-1′), 83.8 (C-2′), 78.7 (C-3′), 72.0 (C-4′), 78.3 (C-5′), 63.0 (C-6′), 106.4 (Glc-II-C-1′′), 77.6 (C-2′′), 78.7 (C-3′′), 72.0 (C-4′′), 78.5 (C-5′′), 63.2 (C-6′′). The structure of compound 4 was identified as ginsenoside Rk_1_ by comparing the NMR data and MS data with reported data.^20^

#### 2.4.5 Ginsenoside Rg_5_ (5)

White amorphous powder; HRESIMS *m*/*z* 789.4569 [M + Na]^+^. ^1^H-NMR (C_5_D_5_N, 500 MHz) *δ*: 1.00 (3H, s, H-18), 0.80 (3H, s, H-19), 1.83 (3H, s, H-21), 5.52 (1H, t, *J* = 6.8 Hz, H-22), 5.25 (1H, t, *J* = 6.8 Hz, H-24), 1.65 (3H, s, H-26), 1.59 (3H, s, H-27), 1.29 (3H, s, H-28), 1.10 (3H, s, H-29), 0.95 (3H, s, H-30), 4.93 (1H, d, *J* = 7.8 Hz, Glc-I-H-1′), 5.37 (1H, d, *J* = 7.7 Hz, Glc-II-H-1′′). ^13^C-NMR (C_5_D_5_N, 125 MHz) *δ*: 39.6(C-1), 28.5(C-2), 89.3(C-3), 40.6(C-4), 56.7(C-5), 18.8(C-6), 35.7(C-7), 40.3(C-8), 51.1(C-9), 37.3(C-10), 33.1(C-11), 72.8(C-12), 50.9(C-13), 51.5(C-14), 32.9(C-15), 31.1(C-16), 51.2(C-17), 16.8(C-18), 17.0(C-19), 140.5(C-20), 13.5(C-21), 123.5(C-22), 27.7(C-23), 123.8(C-24), 131.5(C-25), 26.1(C-26), 18.1(C-27), 28.9(C-28), 16.1(C-29), 17.3(C-30), 105.5 (Glc-I-C-1′), 83.8 (C-2′), 78.7 (C-3′), 72.0 (C-4′), 78.3 (C-5′), 63.0 (C-6′), 106.4 (Glc-II-C-1′′), 77.6 (C-2′′), 78.7 (C-3′′), 72.0 (C-4′′), 78.5 (C-5′′), 63.2 (C-6′′). The structure of compound 5 was identified as ginsenoside Rg_5_ by comparing the NMR data and MS data with reported data.^21^

#### 2.4.6 20(*S*)-ginsenoside Rh_2_ (6)

White amorphous powder; HRESIMS *m*/*z* 1245.8682 [2 M + H]^+^. ^1^H-NMR (C_5_D_5_N, 500 MHz) *δ*: 0.95 (3H, s, H-18), 0.78 (3H, s, H-19), 1.42 (1H, s, H-21), 1.61 (3H, s, H-26), 1.64 (3H, s, H-27), 1.31 (3H, s, H-28), 0.99 (3H, s, H-29), 0.95 (3H, s, H-30), 4.96 (1H, d, *J* = 7.8 Hz, Glc-I-H-1′). ^13^C-NMR (C_5_D_5_N, 125 MHz) *δ*: 39.1(C-1), 26.9(C-2), 88.8(C-3), 39.7(C-4), 56.4(C-5), 18.5(C-6), 35.2(C-7), 40.0(C-8), 50.3(C-9), 37.0(C-10), 32.1(C-11), 71.0(C-12), 48.5(C-13), 51.8(C-14), 31.3(C-15), 26.7(C-16), 54.8(C-17), 16.4(C-18), 15.8(C-19), 73.0(C-20), 27.1(C-21), 35.9(C-22), 23.0(C-23), 126.3(C-24), 130.8(C-25), 25.8(C-26), 17.7(C-27), 28.2(C-28), 16.8(C-29), 17.1(C-30), 107.0 (Glc-C-1′), 75.8 (C-2′), 78.8 (C-3′), 71.9 (C-4′), 78.4 (C-5′), 63.1 (C-6′). The structure of compound 6 was identified as 20(*S*)-ginsenoside Rh_2_ by comparing the NMR data and MS data with reported data.^21^

### 2.5 HPLC analysis of transformation product (1–6)

**Fig. 3:**
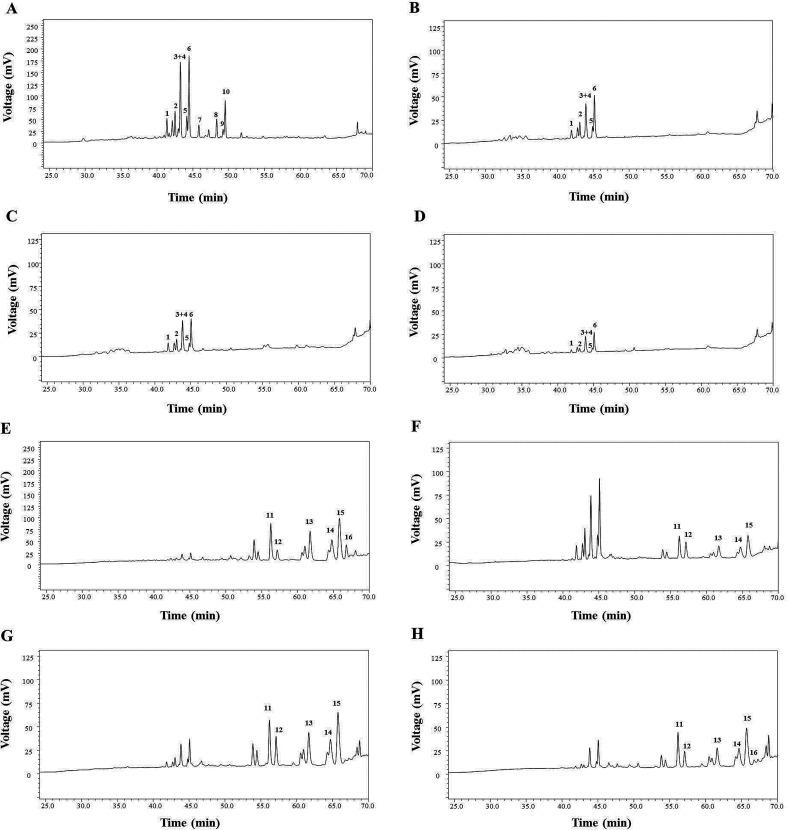
Saponin transformation in different parts of *Panax notoginseng* induced by microwave processing. HPLC chromatograms of the stems and leaves (A), flowers (B), pedicels (C), and seeds (D) of *P. notoginseng*. HPLC chromatograms of the stems and leaves (E), flowers (F), pedicels (G), and seeds (H) of *P. notoginseng* after treatment with microwave processing. Peaks: (1) notoginsenoside Fa; (2) ginsenoside Rb1; (3) ginsenoside Rc; (4) notoginsenoside Fc; (5) ginsenoside Rb2; (6) ginsenoside Rb3; (7) ginsenoside Rd; (8) notoginsenoside Fe; (9) ginsenoside Rd2; (10) gypenoside IX; (11) 20(*S*)-ginsenoside Rg3; (12) 20(*R*)-ginsenoside Rg3; (13) notoginsenoside SFt3; (14) ginsenoside Rk1; (15) ginsenoside Rg5; (16) 20(*S*)-ginsenoside Rh2.

Quantitative analysis of six transformation product (1–6) processed by microwave processing method was determined by high-performance liquid chromatography (HPLC) (Fig. 3). The HPLC data were recorded on a Shimadzu Analytical Instrument (Shimadzu, Kyoto, Japan), equipped with a solvent degasser, a binary pump, an auto sampler and a UV detector. The separation was performed on a VisionHT C18 HL column (250 mm × 4.6 mm, 5 μm, Grace, Deerfield, USA) at the flow rate of 1.0 mL min^−1^. The mobile phase consisted of distilled water (solvent A) and acetonitrile (solvent B) using the following gradient program: 0–20 min, 20–20% B; 20–45 min, 20–46% B; 45–50 min, 46–55% B; 50–66 min, 55–55% B. The detection wavelength and the column temperature were at 203 nm and 35 °C, respectively. The calibration curves for 20(*S*)-Rg_3_ (1), 20(*R*)-Rg_3_ (2), SFt_3_ (3), Rk_1_ (4), Rg_5_ (5) and 20(*S*)-Rh_2_ (6) showed good linearity (*R*^2^ > 0.9990) in the concentration ranges (Table 1). The samples were analyzed by HPLC in three times.

**Table 1:** The results of linear regression of transformation product (1–6) (*n* = 6)

Compounds	Calibration curve	*R* ^2^	Concentration range (μg mL^−1^)
Ginsenoside 20(*S*)-Rg_3_ (1)	*y* = 4 000 000*x* + 9678	0.9996	1.0–1000
Ginsenoside 20(*R*)-Rg_3_(2)	*y* = 4 000 000*x* + 15 317	0.9999	4.0–1000
Notoginsenoside SFt_3_(3)	*y* = 990 240*x*+37 027	0.9999	3.1–3000
Ginsenoside Rk_1_(4)	*y* = 3 000 000*x* + 1465	0.9991	4.0–1000
Ginsenoside Rg_5_(5)	*y* = 758 099*x* + 45 996	0.9997	16.0–5000
Ginsenoside 20(*S*)-Rh_2_(6)	*y* = 3 000 000*x* + 10 426	0.9990	2.5–1000

### 2.6 Anticancer activity *in vitro*

#### 2.6.1 Cell lines and culture

Human cervical cancer (HeLa) and human lung cancer (A549) cell lines were obtained from the Cell Bank of Shanghai Institutes for Biological Sciences, Chinese Academy of Sciences. HeLa cells and A549 cells were cultivated in RPMI Media 1640 (RPMI1640) containing 10% fetal bovine serum, 100 U mL^−1^ penicillin, 0.1 mg mL^−1^ streptomycin and 2 mmol L^−1^l-glutamine. All cultures were maintained with the aeration of 5% CO_2_ at 37 °C.

#### 2.6.2 Cell cytotoxicity assay

The cytotoxicity of EL, MEL and compounds 1–6 on two cancer cell lines was determined by MTT assay.^22^ The cells were seeded in five-well plates (1 × 10^4^ cells per well) and incubated at 37 °C for 24 h. The cells were then treated with EL, MEL, cisplatin and compounds 1–6 at different concentrations (0.1–100 μmol L^−1^). Cisplatin was used as the positive control. After 48 h, 20 μL MTT (5 mg mL^−1^) was added to each well and the cells were incubated for 4 h. The supernatant was removed and then 150 μL of DMSO was added to each well. After the plates were shaken at room temperature for 10 min, the absorbance was measured by PHERA star FSX Microplate Reader (BMG Labtech, Offenburg, Germany) at a wavelength of 570 nm. The half maximal inhibitory concentration (IC_50_) against cancer cells was obtained by curve fitting of a sigmoidal dosage–response curve using nonlinear regression model.

### 2.7 Anticoagulant activity *in vitro*

#### 2.7.1 Animals

Kunming mice (18–22 g) were purchased by Changsha Tianqin Biotechnology Co., China (Qualified no. SCXK 2014-0011). The mice were maintained at a temperature of 25 ± 2 °C, a humidity of 62 ± 2%, and a 12 h dark/light cycle, with unrestricted access to food and water. All animal procedures were performed in accordance with the Guidelines for Care and Use of Laboratory Animals of Kunming University of Science and Technology, and were approved by the Animal Ethics Committee of Kunming University of Science and Technology.

#### 2.7.2 Plasma preparation

The plasma was obtained from mice whole blood mixed with 0.109 mol L^−1^ sodium citrate (9 : 1 ratio of blood to citrate, v/v) by centrifugation at 3000 × *g* for 15 min.

#### 2.7.3 Blood plasma clotting assays

Prothrombin time (PT) was determined with a coagulation analyzer (XN06-IV, Aierfu, Wuhan, China). 100 μL of a solution containing plasma (50 μL) and EL, MEL and compounds 1–6 (50 μL) were incubated at 37 °C for 3 min. The samples of EL and MEL were diluted with purified water to give the following concentrations: 25, 12.5, 5 mg mL^−1^. The concentration of compounds 1–6 is 1 mg mL^−1^. Purified water was used as negative control. The PT assay reagent (100 μL) was added to the mixed samples, and the clotting time was recorded by the analyzer. All clotting tests were performed with three individual replicates.

### 2.8 Statistical analysis

Statistical analyses were performed with SPSS 19.0 software. All data were expressed as mean ± standard deviation (SD). The values of *p* < 0.05 were considered to be statistically significant, and *p* < 0.01 and *p* < 0.001 being very significant.

## 3 Results and discussion

### 3.1 Purification and identification of the transformation products from the stems and leaves of *P. notoginseng* using the microwave processing method

**Fig. 4:**
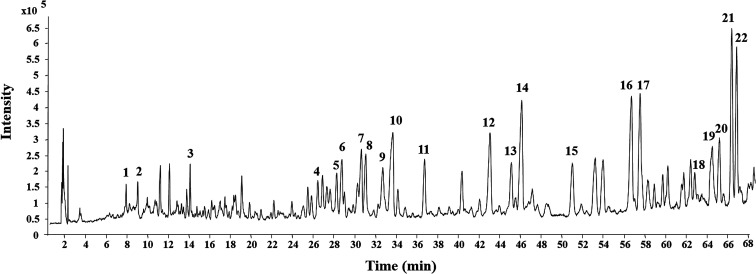
Total ion current chromatogram of microwave processing extract of the stems and leaves of *Panax notoginseng* by UHPLC-ESI-Q-TOF-MS.

The MEL was identified by ultra-high performance liquid chromatography-quantitative time of flight mass spectroscopy (QTOF-MS) in positive mode. The total ion chromatogram of the MEL is shown in Fig. 4. Table 2 summarizes the QTOF-MS data of 22 ginsenosides identified from the MEL. The compounds were determined by the molecular ions based on [M + H]^+^, [M + Na]^+^, or [2 M + H]^+^. The chemical composition of the MEL was mainly 20(*S*)-protopanaxadiol (PPD)-type ginsenosides; the PPD skeleton of which displayed characteristic ions at *m*/*z* 425.^23,24^ Eight transformation products in the MEL were identified as ginsenosides, such as F_2_, 20(*S*)-Rg_3_, 20(*R*)-Rg_3_, SFt_3_, Rk_1_, Rg_5_, 20(*S*)-Rh_2_, and 20(*R*)-Rh_2_. The others had already existed in the PNL before microwave transformation, which were identified by matching molecular formulas with reported data.^3,4^ Six transformation products were purified from the MEL, and their structures were determined by comparing their spectroscopic data with those reported in the literature (Fig. 2). The transformation products were identified as 20(*S*)-ginsenoside Rg_3_ (1),^18^ 20(*R*)-ginsenoside Rg_3_ (2),^18^ notoginsenoside SFt_3_ (3),^19^ ginsenoside Rk_1_ (4),^20^ ginsenoside Rg_5_ (5),^20^ and 20(*S*)-ginsenoside Rh_2_ (6).^21^

**Table 2:** Characterization of ginsenosides in microwave processing extract of stems and leaves of *P. notoginseng* using UHPLC-Q-TOF/MS

No.	Retention time (min)	Experimental *m*/*z*	Molecular formula	Diagnostic fragments (*m*/*z*)	Identification
1	7.31	627.1402 [M + H]^+^	C_42_H_26_O_6_	303.0410, 333.0460, 465.0912, 515.2338, 531.2084, 566.4141, 588.3944	Unknown
2	8.47	611.1461 [M + H]^+^	C_42_H_26_O_5_	287.0467, 449.0962, 566.4133, 588.3948	Unknown
3	13.54	823.4602 [M + Na]^+^	C_42_H_72_O_14_	423.3510, 441.3612, 603.4086, 621.4228, 765.4602	Ginsenoside Rg_1_
4	25.92	1263.6069 [M + Na]^+^	C_59_H_100_O_27_	425.3665, 587.4160, 605.4256, 749.4662, 881.5030	Notoginsenoside Fa
5	27.67	1233.5957 [M + Na]^+^	C_58_H_98_O_26_	425.3659, 603.4095, 749.4646, 823.4584, 881.5069, 955.5007, 1083.5334	Ginsenoside FP_2_
6	28.21	1131.5648 [M + Na]^+^	C_54_H_92_O_23_	425.3658, 587.4171, 749.4646, 929.5196	Ginsenoside Rb_1_
7	30.08	1101.554 [M + Na]^+^	C_53_H_90_O_22_	425.3664, 587.4152, 605.4269, 749.4670, 899.5157	Ginsenoside R_c_
8	30.48	1233.5946 [M + Na]^+^	C_58_H_98_O_26_	425.3666, 587.4158, 605.4267, 749.4656, 881.5080, 916.5333	Notoginsenoside Fc
9	32.15	1101.5561 [M + Na]^+^	C_53_H_90_O_22_	425.3664, 559.2657, 605.4261, 749.4647, 783.4688, 881.5043	Ginsenoside Rb_2_
10	33.01	1101.5591 [M + Na]^+^	C_53_H_90_O_22_	425.3666, 587.4169, 605.4273, 749.4655, 881.5060, 929.5193	Ginsenoside Rb_3_
11	36.19	969.5170 [M + Na]^+^	C_48_H_82_O_18_	425.3663, 587.4159, 767.4730	Ginsenoside R_d_
12	42.46	939.5082 [M + Na]^+^	C_47_H_80_O_17_	425.3672, 587.4170, 605.4255, 719.4548, 737.4652, 749.4659, 767.4736, 793.4525, 881.5041	Notoginsenoside Fe
13	44.55	939.5077 [M + Na]^+^	C_47_H_80_O_17_	425.3673, 587.4156, 605.4274, 719.4553, 737.4644, 749.4686, 767.4758, 785.4860, 881.5065	Ginsenoside Rd_2_
14	45.54	939.5067 [M + Na]^+^	C_47_H_80_O_17_	425.3670, 587.4161, 605.4256, 719.4560, 737.4651, 749.4665, 767.4736, 881.5061	Notoginsenoside Fd
15	50.42	807.4665 [M + Na]^+^	C_42_H_72_O_13_	425.3670, 587.4160, 605.4252, 749.4644, 765.4599	Ginsenoside F_2_
16	56.17	807.4663 [M + Na]^+^	C_42_H_72_O_13_	425.3663, 587.4155, 605.4249, 749.4658	20(*S*)-Ginsenoside Rg_3_
17	57.01	807.4652 [M + Na]^+^	C_42_H_72_O_13_	425.3667, 587.4162, 605.4270, 749.4646	20(*R*)-Ginsenoside Rg_3_
18	61.89	921.4989 [M + Na]^+^	C_47_H_78_O_16_	407.3562, 425.3663, 587.4162, 605.4257, 749.4642, 881.5078	Ginsenoside SFt_3_
19	63.94	789.4595 [M + Na]^+^	C_42_H_70_O_12_	407.3559, 425.3664, 587.4159, 605.4272, 749.4663	Ginsenoside Rk_1_
20	64.67	789.4569 [M + Na]^+^	C_42_H_70_O_12_	407.3563, 425.3663, 587.4163, 605.4263, 749.4657	Ginsenoside Rg_5_
21	65.85	1245.8682 [2M + H]^+^	C_36_H_62_O_8_	407.3562, 425.3663, 587.4156, 605.4254	20(*S*)-Ginsenoside Rh_2_
22	66.34	1245.8688 [2M + H]^+^	C_36_H_62_O_8_	407.3559, 425.3669, 587.4160, 605.4269	20(*R*)-Ginsenoside Rh_2_

### 3.2 Impact of microwave processing conditions on the yields of the transformation products

**Fig. 5:**
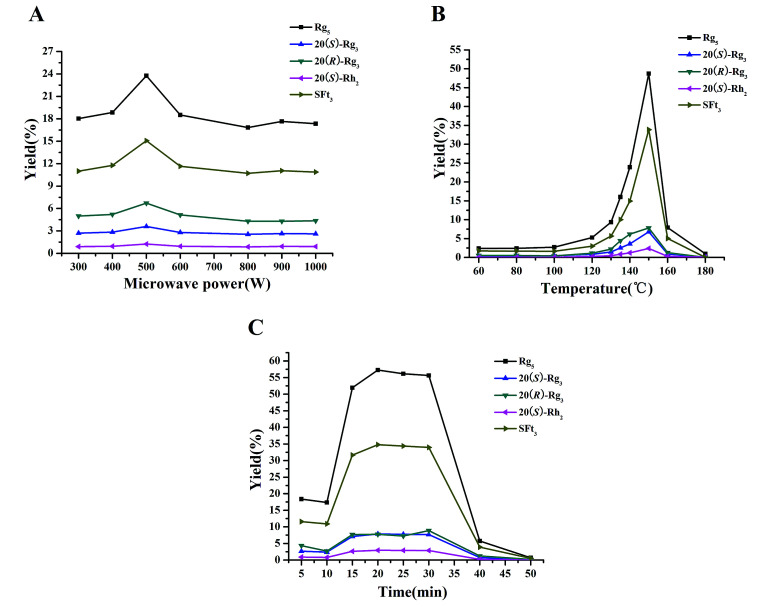
Effects of microwave power (A), temperature (B), and time (C) on yields of transformation products.

Microwave processing as a “green and innovative” technique has been widely used for preservation,^25^ transformation,^13,14^ and extraction^26^ in research and industrial fields. The microwave processing technique has advantages of less time and energy as well as fewer solvents.^16^ In our study, this microwave processing method was applied to degrade the major saponins in the PNL and to produce the rare saponins with fewer sugar moieties. Microwave heating occurs within the molecule, and the glycosidic bonds in the polar molecules break easily.^15^ The major saponins in PNL were hydrolyzed at the glucosyl residue of C-20 to produce ginsenosides 20(*S*)-Rg_3_, 20(*R*)-Rg_3_, 20(*S*)-Rh_2_, Rk_1_, Rg_5_, and notoginsenoside SFt_3_ in the MEL with an increase in microwave power and temperature. The three factors of microwave power, temperature, and time were assessed for yields of the five transformation products by HPLC analysis after microwave processing (Fig. 5).

Microwave power was an important factor in the transformation process. When microwave temperature and time were set to 135 °C and 15 min, microwave power was varied from 300 to 1000 W to evaluate its impact on the yields of the transformation products (Fig. 5A). The highest yield of each transformation product was reached with microwave power of 500 W. The yields of the five transformation products in the MEL were in the order of Rg_5_ > SFt_3_ > 20(*R*)-Rg_3_ > 20(*S*)-Rg_3_ > 20(*S*)-Rh_2_. When the microwave temperature range was from 60–180 °C for 20 min, the yields of the transformation products increased gradually (Fig. 5B). When the temperature was 150 °C, the yields of the transformation products were the highest in the following order: Rg_5_ > SFt_3_ > 20(*R*)-Rg_3_ > 20(*S*)-Rg_3_ > 20(*S*)-Rh_2_. According to the yields of the transformation products, a suitable time for this microwave processing method was 15–30 min (Fig. 5C). The yields of the transformation products were in the following order: Rg_5_ > SFt_3_ > 20(*R*)-Rg_3_ > 20(*S*)-Rg_3_ > 20(*S*)-Rh_2_. It was reported that the contents of the primary degradation products 20(*S*)-Rh_1_, 20(*R*)-Rh_1_, Rk_3_, Rh_4_, 20(*S*)-Rg_3_, 20(*R*)-Rg_3_, Rk_1_, and Rg_5_ for dried notoginseng steamed at 120 °C increase rapidly after 12 h.^12^ A similar yield was achieved in 20 min when the microwave processing method was used. Transformation products are obtained by microwave processing with less energy and less waste compared with the steaming method. According to the yields of the transformation products after microwave processing, the optimum conditions of microwave power, temperature, and time were 500 W, 150 °C, and 20 min, respectively.

### 3.3 Applying the microwave processing method to different parts of *P. notoginseng*

**Fig. 6:**
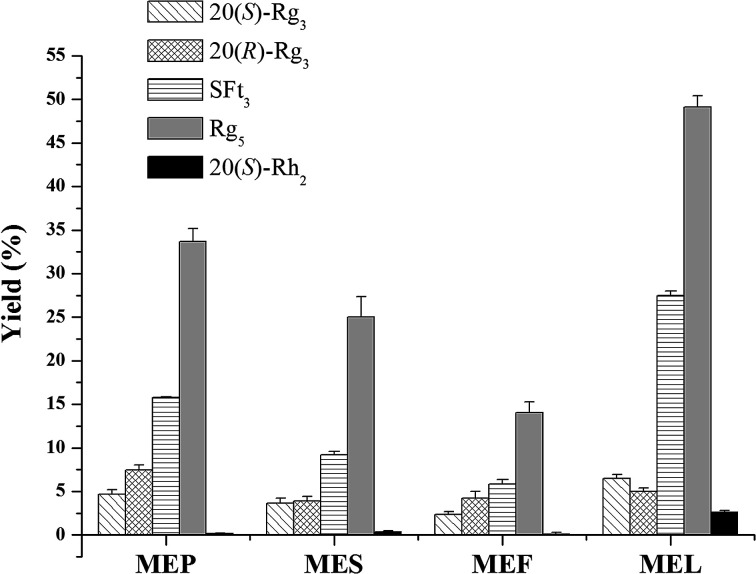
The contents of five transformation products from microwave processing extracts of stems and leaves (MEL), flowers (MEF), seeds (MES), and pedicels (MEP) of *P. notoginseng*. Data are expressed as mean ± SD (*n* = 3).

The results of saponin transformation in different parts of *P. notoginseng* (stems and leaves, flowers, seeds, and pedicels) using microwave processing are shown in Fig. 3 and 6. High performance liquid chromatography chromatograms of stems and leaves, flowers, seeds, and pedicels of *P. notoginseng* revealed similar typical ginsenosides as the PNL, consisting of notoginsenosides Fa and Fc, and ginsenosides Rc, Rb_1_, Rb_2_, and Rb_3_ (Fig. 3A–D). After microwave processing, six newly transformed products of 20(*S*)-Rg_3_, 20(*R*)-Rg_3_, SFt_3_, Rk_1_, Rg_5_, and 20(*S*)-Rh_2_ were generated from the MEL, flowers (MEF), seeds (MES), and pedicels (MEP) of *P. notoginseng* (Fig. 3E–H). As shown in Fig. 6, the Rg_5_ content in the microwave-treated extracts from different parts of *P. notoginseng* was the highest and the Rh_2_ content was the lowest. Under the same microwave processing conditions (500 W, 500 °C, and 20 min) the yields of Rg_5_ in the microwave transformed extracts from different parts of *P. notoginseng* were in the order of: MEL > MEP > MES > MEF. The 20(*S*)-Rh_2_ contents in the MEL and MES were 2.58% and 0.36% respectively, while 20(*S*)-Rh_2_ was not detected in the MEP or MEF. Three special saponins of notoginsenoside Fe, ginsenoside Rd_2_, and gypenoside IX were detected in the PNL (Fig. 3A), which easily lost the glycosyl group at C-20 to generate 20(*S*)-Rh_2_. This result indicates that 20(*S*)-Rh_2_ content in the MEL was much higher than that in the MEP, MES, or MEF.

### 3.4 Transformation mechanism for producing the minor ginsenosides using the microwave processing method

**Fig. 7:**
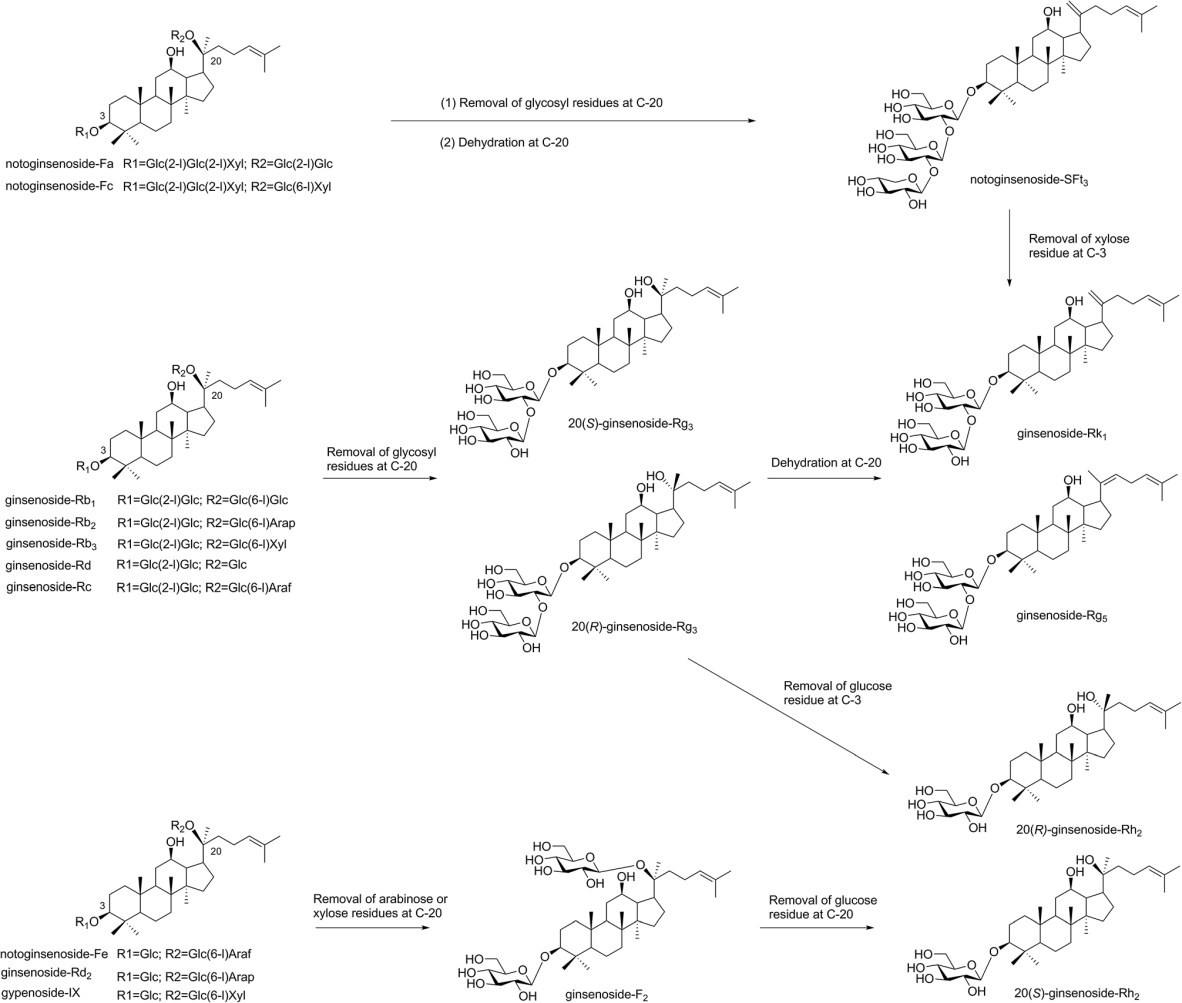
Proposed transformation pathway of saponins in PNL by the microwave processing.

Microwave heating occurs within molecules, which is described as “internal heating”.^15^ If the microwave energy matches with the molecular bond energy, the reactive activity of glycoside bond in the molecule increased and easy to break.^15,27^ In our paper, major saponins lost the glycosyl residues and produced the minor ginsenosides with increased microwave power and temperature. The proposed transformation pathway of saponins in PNL by the microwave processing were shown in Fig. 7. The major saponins, *e.g.*, ginsenosides Rb_1_, Rd, Rc, Rb_2_, and Rb_3_ easily lost the glycosyl residue at C-20 to produce 20(*S*)/(*R*)-Rg_3_, and then formed Rk_1_ and Rg_5_ through dehydration at C-20. Notoginsenosides Fa and Fc were hydrolyzed and dehydrated at C-20 to obtain notoginsenoside SFt_3_. When the microwave temperature was increased, a small amount of 20(*S*)/(*R*)-Rg_3_ was converted to 20(*S*)/(*R*)-Rh_2_ through loss of a glucosyl group at C-3. In addition, the three compounds of notoginsenoside Fe, ginsenoside Rd_2_ and gypenoside IX firstly removed the arabinose or xylose residues at C-3 to produce ginsenoside F_2_, and continued to lose one glucose residue at C-20 to obtain 20(*S*)-Rh_2_. This indicated that the content of 20(*S*)-Rh_2_ was much higher than that of 20(*R*)-Rh_2_ in the microwave processing. When the glycosyl residues at C-20 were lost, 20(*S*/*R*) epimers such as 20(*S*/*R*)-Rg_3_ and 20(*S*/*R*)-Rh_2_ were obtained by the selective attack of the OH group.^28^

The structures of both 20(*S*/*R*)-Rg_3_ and 20(*S*)-Rh_2_ had been characterized with NMR. The ^13^C NMR analysis suggested the obvious changes of the chemical shifts in 20(*S*/*R*) epimers at C-17, C-21 and C-22. The chemical shifts of 20(*S*)-Rg_3_ were *δ*_C_ 55.2(C-17), 27.5(C-21) and 36.3(C-22) ppm, while those of 20(*R*)-Rg_3_ were *δ*_C_ 50.6(C-17), 22.8(C-21) and 43.2(C-22) ppm. The values in 20(*S*)-Rh_2_ were also observed in the peaks of *δ*_C_ 54.8(C-17), 27.1(C-21) and 35.9(C-22) ppm. The results were in agreement with the reported data.^18^ After dehydration at Δ20(21) or Δ20(22), the values were shifted to *δ*_C_ 155.75 ± 0.15(C-20) and 108.35 ± 0.15(C-21) ppm in SFt_3_ and Rk_1_, and *δ*_C_ 140.5(C-20) and 123.5(C-22) ppm in Rg_5_, respectively.

### 3.5 Antiproliferative effect of the transformation products on the cancer cell lines

**Fig. 8:**
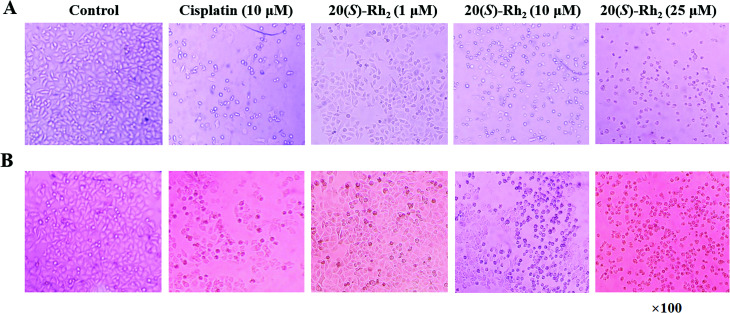
20(*S*)-Ginsenoside Rh2 inhibits cell proliferation of human cervical cancer Hela (A) and human lung cancer A549 (B). The cells were treated with 20(*S*)-ginsenoside Rh2 at different concentrations.

The antiproliferative activities of the PNL extract before and after microwave processing, and transformation products 1–6 were evaluated in two human cancer cell lines (HeLa and A549) by the MTT assay. The results are expressed as the IC_50_ of the half maximal inhibitory concentration against cancer cells. As shown in Table 3, the extract of stems and leaves of *P. notoginseng* (EL) had no inhibitory effect on human cervical cancer (Hela) or human lung cancer (A549), and no IC_50_ values were obtained. After the EL microwave treatment, microwave processing of the MEL resulted in better inhibitory activity against Hela and A549 cells, with IC_50_ values of 42.97 and 50.47 μg mL^−1^, respectively. Microwave processing of ginseng enhances its anticancer activity due to the increased content of the ginsenosides Rg_3_, Rg_5_, and Rk_1_.^13,14^ Further research showed that the transformation products of ginsenosides 20(*S*)-Rg_3_, 20(*R*)-Rg_3_, Rk_1_, Rg_5_, and 20(*S*)-Rh_2_ and notoginsenoside SFt_3_ in the MEL actively inhibited the Hela and A549 cells. Among compounds 1–6, ginsenoside 20(*S*)-Rh_2_ (6) displayed lower IC_50_ values (8.23 and 12.45 μM) than cisplatin (8.43 and 16.85 μM) in Hela and A549 cells, respectively. The ginsenoside 20(*S*)-Rh_2_ effectively inhibits tumor cell growth and survival in both animal models and cell lines.^29–31^ 20(*S*)-Rh_2_ directly binds to Annexin A_2_, which promotes apoptosis by inhibiting NF-kB activity and down-regulating anti-apoptosis gene expression.^31^ To confirm the antiproliferative effect of the transformation products, the morphologies in Hela and A549 cells treated with ginsenoside 20(*S*)-Rh_2_ was observed under a microscope (Fig. 8). In the treatment of Hela and A549 cells, 20(*S*)-Rh_2_ at the concentration of 1 μM had no significant effect on cell morphology. While increasing concentration to 10 or 25 μM, 20(*S*)-Rh_2_ induced cell body shrinkage and death in Hela and A549 cells.

**Table 3:** Antiproliferative effect of the extracts (EL and MEL, μg mL^−1^) and compounds 1–6 (μM) in human cancer cell lines^a^

Compounds	IC_50_ values	
	Hela	A549
20(*S*)-Ginsenoside Rg_3_ (1)	23.96	20.87
20(*R*)-Ginsenoside Rg_3_ (2)	24.87	28.47
Notoginsenoside SFt_3_ (3)	19.67	21.48
Ginsenoside Rk_1_ (4)	11.97	9.52
Ginsenoside Rg_5_ (5)	42.96	45.24
20(*S*)-Ginsenoside Rh_2_ (6)	8.23	12.45
Cisplatin	8.43	16.85
MEL	42.97	50.47
EL	>200	>200

a Hela: human cervical cancer. A549: human lung cancer. IC_50_: concentration that inhibits 50% of cell growth. EL: extract of stems and leaves of *P. notoginseng*. MEL: microwave processing extract of stems and leaves of *P. notoginseng*.

### 3.6 The anticoagulant effect of the transformation products *in vitro*

**Fig. 9:**
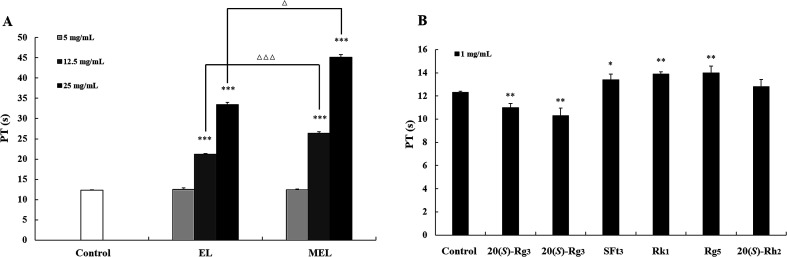
Anticoagulant activities of raw and microwave processing extracts of the stems and leaves of *P. notoginseng*. (A) PT assays of the extract of stems and leaves of *P. notoginseng* (EL) and microwave processing extract of stems and leaves of *P. notoginseng* (MEL). (B) PT assays of ginsenosides 20(*S*)-Rg3, 20(*R*)-Rg3, Rk1, Rg5, 20(*S*)-Rh2, and notoginsenoside SFt3. Data are expressed as mean ± SD (*n* = 3). Compared with the negative control group: *p < 0.05, **p < 0.01, ***p < 0.001. EL group compared with MEL group: Δ*p* < 0.05, ΔΔ*p* < 0.01, ΔΔΔ*p* < 0.001.

According to previous reports, different parts of *P. notoginseng* have anticoagulant activity, and the leaves possess stronger anticoagulant activity among them.^32^ The components of the PNL changed dramatically after the microwave processing treatment (Fig. 3), which may have resulted in the change in anticoagulant activity. Therefore, the effects of the EL, MEL and transformation products 1–6 on anticoagulation were evaluated by the PT assay (Fig. 9). The PT value reflects the activity of the coagulation system *in vitro*.^33^ As shown in Fig. 9A, the EL and MEL had significantly prolonged clotting times in a concentration-dependent manner (*p* < 0.001). The anticoagulant activity of the MEL was better than that of the EL after comparing the extracts of the PNL before and after the microwave treatment. Further studies showed that the degradation products of ginsenosides Rk_1_ and Rg_5_, as well as notoginsenoside SFt_3_ prolonged coagulation time, while ginsenosides 20 (*S*/*R*)-Rg_3_ shortened coagulation time (Fig. 9B). The structure–activity results revealed that the hydroxy-substituted compounds at C-20 (20 (*S*/*R*)-Rg_3_) had the hemostatic activities, while Rk_1_, SFt_3_ and Rg_5_ with double bond at Δ20(21) or Δ20(22) played the anticoagulant activities. The anticoagulant activity of the MEL increased due to increases in the concentrations of Rk_1_, Rg_5_, and SFt_3_. This result was consistent with previous findings that ginsenosides Rk_1_ and Rg_5_ inhibit arachidonic acid-induced platelet aggregation in a dose dependent manner.^34^

## 4 Conclusion

In summary, microwave processing was applied to produce rare saponins in the PNL. Six transformation products of ginsenosides 20(*S*)-Rg_3_, 20(*R*)-Rg_3_, Rk_1_, Rg_5_, 20(*S*)-Rh_2_, and notoginsenoside SFt_3_ were isolated from the MEL. Microwave power, temperature, and time affected the yields of the six transformation products. This transformation method was widely applied for producing the minor ginsenosides in stems and leaves, flowers, seeds and pedicels of *P. notoginseng*. The MEL had an obvious inhibitory effect on human cervical cancer and human lung cancer cell lines. The MEL had a stronger anticoagulant effect due to increased contents of ginsenosides Rk_1_, Rg_5_, and notoginsenoside SFt_3_. Therefore, the PNL may be a good source of anticancer and anticoagulant therapeutics after microwave processing.

## Conflicts of interest

All authors declare no conflicts of interest.

## Supplementary Material

## References

[cit1] Qu Y., Liu Y., Huang L. Q., Guo L. P., Cui X. M. (2014). China J. Chin. Mater. Med..

[cit2] Zhou J. M., Cui X. M., Zhang W. B., Zeng H. C., Ma N. (2009). Res. Pract. Chin. Med..

[cit3] Liu F., Ma N., He C. W., Hu Y. J., Li P. M., Chen W., Su H. X., Wan J. B. (2018). J. Ginseng Res..

[cit4] Mao Q., Yang J., Cui X. M., Li J. J., Qi Y. T., Zhang P. H., Wang Q. (2012). J. Pharm. Biomed. Anal..

[cit5] Wang T., Guo R. X., Zhou G. H., Zhou X. D., Kou Z. Z., Sui F., Li C., Tang L. Y., Wang Z. J. (2016). J. Ethnopharmacol..

[cit6] Sun M., Ye Y., Xiao L., Duan X., Zhang Y., Zhang H. (2017). Int. J. Mol. Med..

[cit7] Peng M., Yi Y. X., Zhang T., Ding Y., Le J. (2018). Front. Pharmacol..

[cit8] Lu P., Su W., Miao Z. H., Niu H. R., Liu J., Hua Q. L. (2008). Chin. J. Integr. Med..

[cit9] Wang R. F., Li J., Hu H. J., Li J., Yang Y. B., Yang L., Wang Z. T. (2018). J. Ginseng Res..

[cit10] Choi H. S., Kim S. Y., Park Y., Jung E. Y., Suh H. J. (2014). J. Ginseng Res..

[cit11] Cui L., Wu S. Q., Zhao C. A., Yin C. R. (2016). J. Ginseng Res..

[cit12] Wang D., Liao P. Y., Zhu H. T., Chen K. K., Xu M., Zhang Y. J., Yang C. R. (2012). Food Chem..

[cit13] Choi P., Park J. Y., Kim T., Park S. H., Kim H. K., Kang K. S., Ham J. (2015). J. Funct. Foods.

[cit14] Park J. Y., Choi P., Kim H. K., Kang K. S., Ham J. (2016). J. Ginseng Res..

[cit15] Galema S. A. (1997). Chem. Soc. Rev..

[cit16] Chemat F., Rombaut N., Meullemiestre A., Turk M., Perino S., Fabiano-Tixier A. S., Abert-Vian M. (2017). Innovative Food Sci. Emerging Technol..

[cit17] Liu H. Y., Pan J., Yang Y., Cui X. M., Qu Y. (2018). Molecules.

[cit18] Yang H., Kim J. Y., Kim S. O., Yoo Y. H., Sung S. H. (2014). J. Ginseng Res..

[cit19] Liu Q., Lv J. J., Xu M., Wang D., Zhu H. T., Yang C. R., Zhang Y. J. (2011). Nat. Prod.
Bioprospect..

[cit20] Meng J., Duan W. W. (2017). West China J. Pharm. Sci..

[cit21] Li H. Z., Zhang Y. J., Yang C. R. (2006). Nat. Prod. Res. Dev..

[cit22] Shen Q. K., Chen Z. A., Zhang H. J., Li J. L., Liu C. F., Gong G. H., Quan Z. S. (2018). J. Enzyme Inhib. Med. Chem..

[cit23] Geng C., Yin J. Y., Yu X. H., Yang Y. X., Liu J. Y., Sun D. D., Chen F. B., Wei Z. L., Meng Q., Liu J. H. (2015). Rapid Commun. Mass Spectrom..

[cit24] Zhang X. R., Zhang J., Li W., Liu L., Sun B. S., Guo Z. H., Shi C. H., Zhao Y. Q. (2014). PLoS One.

[cit25] Gowen A., Abu-Ghannam N., Frias J., Oliveira J. (2006). Trends Food Sci. Technol..

[cit26] Jacotet-Navarro M., Rombaut N., Deslis S., Fabiano-Tixier A. S., Pierre F. X., Bily A., Chemat F. (2016). Green Chem..

[cit27] Shao J., Yang Y., Zhong Q. (2003). Polym. Degrad. Stab..

[cit28] Kang K. S., Yamabe N., Kim H. Y., Okamoto T., Sei Y., Yokozawa T. (2007). J. Ethnopharmacol..

[cit29] Shi Q., Li J., Feng Z., Zhao L., Luo L., You Z., Li D., Xia J., Zou G., Chen D. (2014). Mol. Med. Rep..

[cit30] Li B., Zhao J., Wang C. Z., Searle J., He T. C., Yuan C. S., Du W. (2011). Cancer Lett..

[cit31] Wang Y. S., Lin Y. J., Li H., Li Y., Song Z. G., Jin Y. H. (2017). Sci. Rep..

[cit32] Zhao Y. L., Fan K., Liu Y., Liang Z. S., Han R. L. (2013). Acta Bot. Boreali-Occident. Sin..

[cit33] Li C. T., Wang H. B., Xu B. J. (2013). Pharm. Biol..

[cit34] Lee J. G., Lee Y. Y., Kim S. Y., Pyo J. S., Yun-Choi H. S., Park J. H. (2009). Die Pharmazie.

